# YeATSAM analysis of the walnut and chickpea transcriptome reveals key genes undetected by current annotation tools

**DOI:** 10.12688/f1000research.10040.1

**Published:** 2016-11-17

**Authors:** Sandeep Chakraborty, Pedro J. Martínez-García, Abhaya M. Dandekar

**Affiliations:** 1 Department of Plant Sciences, University of California, Davis, USA

**Keywords:** RNA-seq, transcriptome, MAKER-P, genome annotation, berberine bridge enzyme, Trinity, walnut genome sequence

## Abstract

**Background**: The transcriptome, a treasure trove of gene space information, remains severely under-used by current genome annotation methods. **Methods**: Here, we present an annotation method in the YeATS suite (YeATSAM), based on information encoded by the transcriptome, that demonstrates artifacts of the assembler, which must be addressed to achieve proper annotation. **Results and Discussion: **YeATSAM was applied to the transcriptome obtained from twenty walnut tissues and compared to MAKER-P annotation of the recently published walnut genome sequence (WGS). MAKER-P and YeATSAM both failed to annotate several hundred proteins found by the other. Although many of these unannotated proteins have repetitive sequences (possibly transposable elements), other crucial proteins were excluded by each method. An egg cell-secreted protein and a homer protein were undetected by YeATSAM, although these did not produce any transcripts. Importantly, MAKER-P failed to classify key photosynthesis-related proteins, which we show emanated from Trinity assembly artifacts potentially not handled by MAKER-P. Also, no proteins from the large berberine bridge enzyme (BBE) family were annotated by MAKER-P. BBE is implicated in biosynthesis of several alkaloids metabolites, like anti-microbial berberine. As further validation, YeATSAM identified ~1000 genes that are not annotated in the NCBI database by Gnomon. YeATSAM used a RNA-seq derived chickpea ( *Cicer arietinum* L.) transcriptome assembled using Newbler v2.3. **Conclusions: **Since the current version of YeATSAM does not have an *ab initio* module, we suggest a combined annotation scheme using both MAKER-P and YeATSAM to comprehensively and accurately annotate the WGS.

## Introduction

The genome of a particular organism is static in all cells, unlike the dynamic transcriptome, which is the transcription of the gene space into RNA molecules in a fashion responsive to a variety of factors, such as developmental stage, tissue, and external stimuli. RNA-seq, a high-throughput RNA sequencing method, has radically transformed the identification of transcripts and quantification of transcriptional levels ( Flintoft, 2008; Wang *et al.*, 2009). It is supported by a diverse set of computational methods for analyzing the resulting data ( Chakraborty *et al.*, 2015; Chang *et al.*, 2015; Chu *et al.*, 2013; Fu *et al.*, 2012; Grabherr *et al.*, 2011; Lohse *et al.*, 2012; Mbandi *et al.*, 2015; Schulz *et al.*, 2012; Simpson *et al.*, 2009; Trapnell *et al.*, 2009; Trapnell *et al.*, 2012; Wang *et al.*, 2010; Zerbino & Birney, 2008).

Rapid advances in genome sequencing technologies have generated sequences for a deluge of organisms and species. The task of annotating these sequences has been addressed by several flows. These pipelines are categorized in http://omictools.com/genome-annotation-category and http://genometools.org/ and reviewed in ( Yandell & Ence, 2012). Here, we focus specifically on MAKER-P ( Campbell *et al.*, 2014; Holt & Yandell, 2011; Law *et al.*, 2015; Neale *et al.*, 2014), which was used to annotate the recently published walnut genome sequence (WGS) ( Martínez-García *et al.*, 2016).

In the current study, the YeATS suite ( Chakraborty *et al.*, 2015) was enhanced to include genome annotation capabilities using RNA-seq-derived transcriptomes (YeATS **a**nnotation module - YeATSAM). First, the Trinity-assembled transcriptome obtained from twenty different tissues was compared to the WGS, excluding transcripts emanating from extraneous sources. This step incidentally revealed both biodiversity and plant-microbe interactions in walnut tree(s) from Davis, California ( Chakraborty *et al.*, 2016a). The WGS-derived transcripts were split into three open reading frames (ORFs), which were subjected to BLAST analysis using a plant proteome database obtained from the Ensembl database ( Kersey *et al.*, 2016). Transcripts can contain more than one significant ORF and must be handled differently depending on whether they map to the same or a different protein. The resulting analysis provided the WGS annotation.

Both MAKER-P and YeATSAM failed to annotate several hundred proteins annotated by the other. Many of the proteins had repetitive sequences or domains that, although difficult to detect, do not represent critical proteins during annotation. An egg cell-secreted protein ( Sprunck *et al.*, 2012), a copper chaperone ( Shin *et al.*, 2012), and a clavata3/ESR-Related protein ( Kinoshita *et al.*, 2007) were among the proteins not detected through the YeATSAM flow. Some proteins undetected in the MAKER-P flow are more significant in the context of a plant genome: several photosynthesis-related proteins encoded by the chloroplast ( Nelson & Yocum, 2006) and the large family of FAD-binding berberine bridge enzymes (BBE) involved in biosynthesis of antimicrobial benzophenanthridines ( Cheney, 1963; Winkler *et al.*, 2008). We posited possible reasons for such exclusions and recommend incorporating both flows for comprehensive enumeration of genes in the WGS.

As further validation, YeATSAM was applied to chickpea ( *Cicer arietinum* L.), an important pulse crop with many nutritional and health benefits ( Jukanti *et al.*, 2012). The RNA-seq-derived transcriptome of chickpea has also been sequenced ( Garg *et al.*, 2011) and was processed through the YeATSAM pipeline to identify ~1000 proteins that are encoded by these transcripts, but are not annotated in the NCBI database, most of which were annotated using Gnomon ( Souvorov *et al.*, 2010).

## Methods

**Figure 1.**
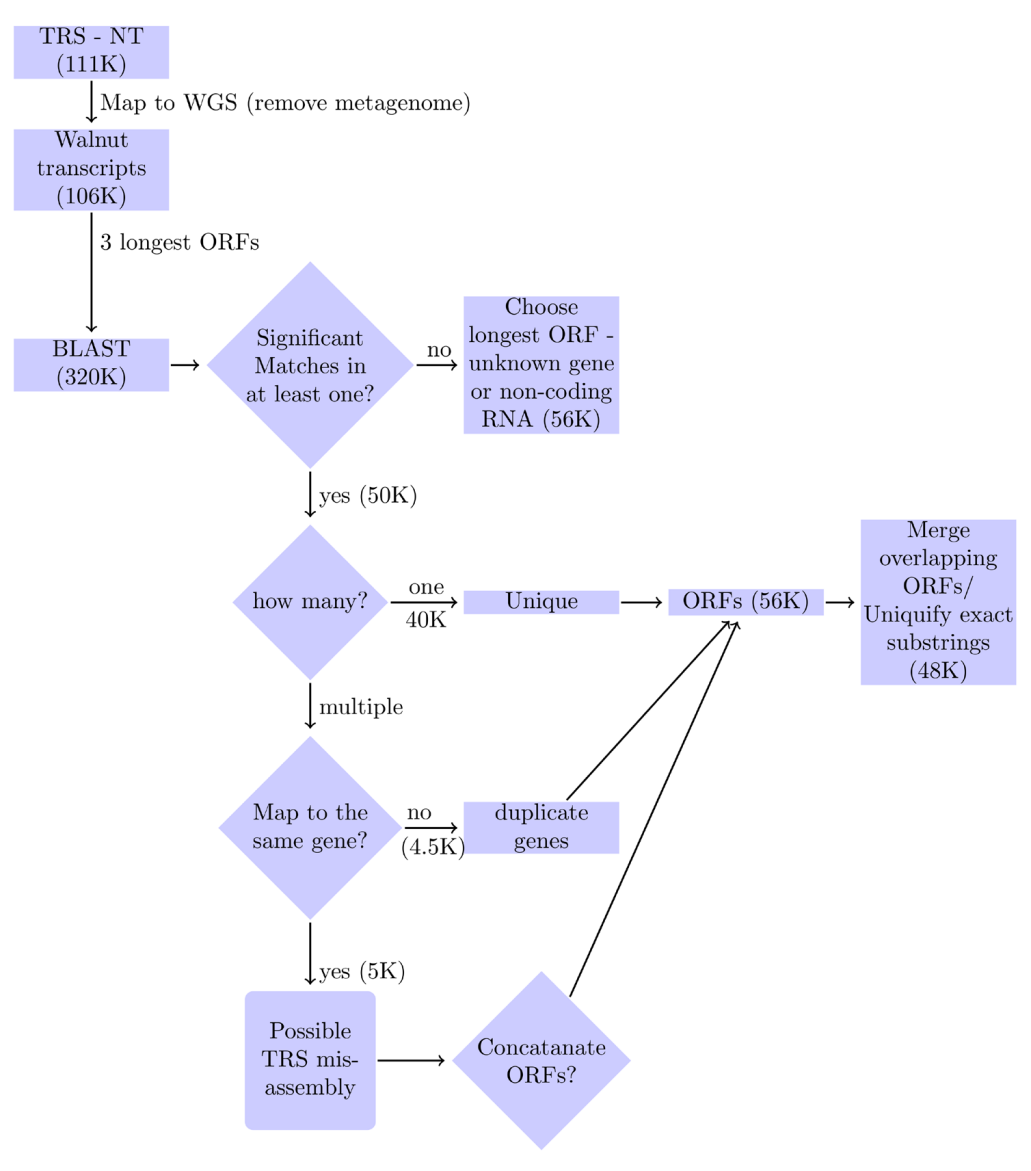
YeATSAM flow. First, transcripts from extraneous organisms are pruned. Next, the three longest open reading frames (ORFs) from each transcript undergo BLAST analysis to a database of plant peptides. Depending on the number of significant matches, the transcripts are clustered as: ( **a**) None - either a previously unknown gene, or non-coding RNA. ( **b**) One - Unique ORF ( **c**) Multiple ORFs matching to the same gene - merge the ORFs if the Evalue of the combined ORF is significantly lower. ( **d**) Multiple ORFs matching to different genes - duplicate the transcripts, associating each with a different ORF. Subsequently, the ORFs are merged based on overlapping amino acid sequences and exact substrings are removed.

The input to YeATSAM is a set of post-assembly transcripts (∅ _*TRS*_) and the walnut genome sequence (WGS) ( Figure 1). Transcripts that do not align to the WGS were removed ( Chakraborty *et al.*, 2016a). A BLAST database of protein peptides (plantpep.fasta: 1M seqeunces) using ~30 organisms (list.plants) from the Ensembl genome was created ( Kersey *et al.*, 2016). The three longest open reading frames (ORF), obtained using the ‘getorf’ utility in the EMBOSS suite ( Rice *et al.*, 2000), for each transcript in (∅ _*TRS*_) underwent BLAST analysis ( Camacho *et al.*, 2013) to the ‘plantpep.fasta’. For cutoff E-value=1E-8, depending on the number of matches, the transcripts were clustered as:

1. None - either a previously unknown gene or non-coding RNA.
2. One - unique ORF.
3. Multiple ORFs matching to the same gene - merge the ORFs if the Evalue of the combined ORF is significantly lower.
4. Multiple ORFs matching to different genes - duplicate the transcripts, associating each transcript with a different ORF.

### *In vitro* methods

Fifteen samples of walnut tissue were gathered from Chandler trees growing in the Stuke block at UC Davis between April and October 2008. Four additional samples were taken from Chandler plant material from the same orchard maintained in tissue culture. Several grams of leaf and root tissue from each plant were frozen in liquid nitrogen and then transferred to a -80 C freezer. RNA was isolated from each sample using the hot borate method ( Wilkins & Smart, 1996) followed by purification and DNAse treatment using an RNA/DNA Mini Kit (Qiagen, Valencia, CA) per the manufacturer’s protocol. High-quality RNA was confirmed by running an aliquot of each sample on an Experion Automated Electrophoresis System (Bio-Rad Laboratories, Hercules, CA). The cDNA libraries were constructed following the Illumina mRNA-sequencing sample preparation protocol (Illumina Inc., San Diego, CA). Final elution was performed with 16µL RNase-free water. The quality of each library was determined using a BioRad Experion (BioRad, Hercules, CA). Each library was run as an independent lane on a Genome Analyzer II (Illumina, San Diego, CA) to generate 85bp paired-end sequences from each cDNA library. Over a billion reads were obtained. Prior to assembly, all reads underwent quality control for paired-end reads and trimming using Sickle v1.33 ( Joshi & Fass, 2011). The minimum read length was 45bp with a minimum Sanger quality score of 35. The quality-controlled reads were *de novo* assembled with Trinity v2.0.6 ( Grabherr *et al.*, 2011). Standard parameters were used and the minimum contig length was 300bp. Individual assemblies for each library and a combined assembly of all tissues were performed.

The walnut genome sequence has been released to the public domain ( http://ucanr.edu/sites/wgig/). The Illumina (Genome Analyzer II) for all 20 tissues can be accessed at http://www.ncbi.nlm.nih.gov/sra/PRJNA232394.

The transcriptome of *Cicer arietinum* (transHybrid.fasta, ICC4958; Desi chickpea) was obtained from http://www.nipgr.res.in/ctdb.html ( Garg *et al.*, 2011). The dataset ‘represents optimized *de novo* hybrid assembly of 454 and short-read sequence data.’ About two million 454 reads were assembled using Newbler v2.3 followed by hybrid assembly with 53409 transcripts generated by optimized short-read data assembly using TGICL, as reported previously ( Garg *et al.*, 2011). The set of annotated proteins from chickpea was obtained from the NCBI database (chickpea.pep.fasta, N=34198).

PHYML v3.0 was used to generate phylogenetic trees from alignments ( Guindon *et al.*, 2005). Multiple sequence alignment was done using ClustalW ( Larkin *et al.*, 2007) and figures were generated using the ENDscript server 2.0 ( Robert & Gouet, 2014). The source code written in Perl is provided as Dataset 1 (YeATSAM.tgz). A README is provided inside the top-level directory for installation and running the programs.

## Results and discussion

The input to YeATSAM was ~111K Trinity-assembled transcripts (Combined TrinityFull.fasta) ( Figure 1). Each transcript was aligned to the WGS (wgs.5d.scafSeq200+.trimmed) using BLAST ( Camacho *et al.*, 2013). Transcripts that did not align to the WGS (cutoff BLAST bitscore=75) were excluded ( Chakraborty *et al.*, 2016a). Those transcripts that aligned to the WGS (list.transcriptome.clean: 106K) were split into the three longest open reading frames (ORF) (list.transcriptome.clean.ORFS: 320K).

A BLAST database of protein peptides (plantpep.fasta:1M sequences) using ~30 organisms (list.plants) from the Ensembl genome was created ( Kersey *et al.*, 2016). The availability of proteomes from related organisms accelerates the annotation. The BLAST results of list.transcriptome.clean.ORFS: 320K on ‘plantpep.fasta’ was processed using a cutoff: bitscore=60, Evalue~=1E-10.

### Merging ORFs: broken transcripts

**Figure 2.**
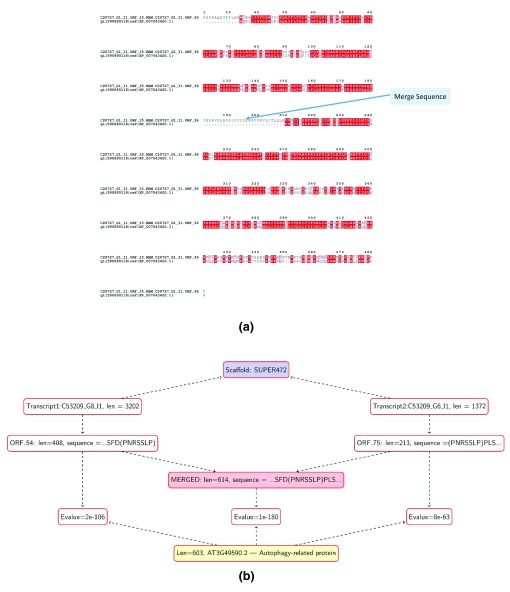
Open reading frames (ORF) that can be merged. ( **a**) ORFs from the same transcript: C20727_G1_I1 has two ORFS (ORF 15 and ORF 36) matching to a DNA repair metallo-β-lactamase family protein (Accession number: XP007043420.1) with high significance. We merged the two ORFs (inserting ‘ZZZ’) since the Evalue of the combined ORF is significantly reduced. ( **b**) ORFs from different transcripts: We merged ORFs from two different transcripts (C53209_G8_I1 and C53209_G6_I1), since both transcripts map to the same scaffold (SUPER472) can be overlapped based on the sequence string ‘PNRSSLP’, and the merged ORF has a significantly reduced Evalue.

There are two instances in which ORFs can be merged to create a longer amino acid sequence. The first scenario occurs when a particular transcript has multiple ORFs that match to the same protein with high significance, indicating that a sequencing or assembly error has broken a contiguous ORF ( Chakraborty *et al.*, 2015). In total, 5% of the present transcripts (5,000 of 106,000) had two or more ORFs matching with high significance to the same protein, exactly mirroring the 5% error rates seen in transcripts restricted to the transcriptome from the tissue at the heartwood/sapwood transition zone in black walnut ( Chakraborty *et al.*, 2015). While most of these transcripts have repetitive elements, there were other non-repetitive sequences with this particular problem. C20727_G1_I1 is one example: it has two ORFS, ORF_15 and ORF_36, that match a DNA repair metallo-β-lactamase family protein (Accession number: XP007043420.1) with Evalues=9E-70 and 6E-96, respectively ( Figure 2a). The two ORFs were merged (inserting the sequence ‘ZZZ’, although the length of the missing fragment is not known) since the Evalue of the combined ORF reduces to 2E-175 and the merged sequence was chosen as representative for the transcript. ORFs are not merged when the combined ORF did not significantly decrease the Evalue and the longer ORF was selected to represent the transcript.

The other scenario occurs when the assembler fails to merge two transcripts into a single one. In this instance, two ORFs emanating from different transcripts with significant overlaps were merged. While the merging of two ORFs was described previously ( Chakraborty *et al.*, 2015), we introduced an additional filter to select mergeable ORFs based on whether the E-value obtained by merging the two ORFs is significantly reduced. For example, transcripts C53209_G8_I1 and C53209_G6_I1 both map to the scaffold SUPER472 and their corresponding ORFs can be merged based on the sequence string ‘PNRSSLP’ ( Figure 2b). The individual ORFs and the combined ORFs align to an autophagy-related protein (TAIR ID: AT3G49590.2) with Evalues 2e-106, 8e-63, and 1e-180, respectively. The increased significance of the combined ORF, in addition to other checks, like ensuring that mapping is to the same scaffold, adds further support to the fact that these transcripts should have been contiguous in the final assembled transcriptome.

### Transcripts with multiple ORFs

About 3% of transcripts have ORFs that map to different proteins. Some transcripts should not have been merged. C1089_G1_I1 is an interesting example: a 4574 nt transcript that maps to the chloroplast and encodes two genes. One is highly variable and the other is conserved. The two ORFS, ORF_64 (fwd: 1117-2631) and ORF_108 (fwd: 3195 - 4271), map to maturase K (TAIR ID: ATCG00040.1) and photosystem II reaction center protein (TAIR ID: ATCG00020.1) with very high significance. Maturase K is a good candidate for barcoding angiosperms because it has highly variable coding sequences ( Yu *et al.*, 2011), while the photosystem II reaction center protein is completely conserved (100% similarity with *Arabidopsis*). Another example is C19241_G1_I1 (4702 nt), split into ORF_68 (fwd: 176-3487) and ORF_115 (reverse: 4509-4096) encoding a damaged DNA binding protein (TAIR ID: AT4G05420.1) and photosystem I subunit K (TAIR ID: AT1G30380.1) with high significance, respectively. These transcripts are split in the YeATSAM flow, resulting in one ORF per transcript. Subsequently, this artifact of the Trinity assembly led to several unannotated proteins in the MAKER-P flow.

### Identifying genes not detected by either YeATSAM or MAKER-P

We compared the annotations of walnut by MAKER-P (walnut.wgs.5d.all.maker.proteins.fasta) and YeATSAM (DB.ORFBEST.60). MAKER-P and YeATSAM each failed to annotate several proteins identified by the other (MAKER-P=~4000; YeATSAM=700). Although most of these unannotated proteins have repetitive sequences (transposable elements), some vital, non-repetitive proteins were excluded by each method. For example, an egg cell-secreted protein (‘WALNUT 00001389-RA’) ( Sprunck *et al.*, 2012), a Clavata3/esr-related gene (‘WALNUT 00023705-RA’) ( Kinoshita *et al.*, 2007) and a copper chaperone (‘WALNUT 00006344-RA’) ( Shin *et al.*, 2012) were not annotated in the YeATSAM flow. These genes do not have transcripts in the twenty tissues analyzed in the current study and are most likely pseudogenes.

### Proteins unannotated by MAKER-P

MAKER-P fails to annotate many key photosystem-related proteins ( Table 1). The transcript C59245_G1_I1 has ORF_43 (fwd: 176-1714) and ORF_70 (fwd: 2212-2496) mapping to photosystem II reaction center protein B (PSBB) and photosystem II reaction center protein H (PSBH), respectively. While MAKER-P does annotate PSBB, it failed to detect PSBH. These proteins map to transcripts encoding two significant ORFs (>1E-10), indicating that failure to handle this might have excluded these proteins. Also, these proteins are encoded by the chloroplast. However, this limitation of MAKER-P is not confined to transcripts emanating from the chloroplast. For example, C48031_G3_I1 encodes a leucine-rich repeat transmembrane protein kinase (AT5G48940.1) and a metallo- *β*-lactamase family protein (TAIR ID: AT4G33540.1) and is mapped to scaffold ‘SUPER374’. MAKER-P failed to annotate the β-lactamase family protein.

**Table 1.** Key photosystem-related proteins in the chloroplast not annotated by MAKER-P and YeATSAM. These transcripts have multiple open reading frames (ORFs) mapping to different proteins with high significance. For example, C59245_G1_I1 has another ORF (43) which maps to photosystem II reaction center protein B (PSBB). MAKER-P annotates PSBB, but not PSBH. These transcripts all emanate from the chloroplast, although not all genes that MAKER-P failed to annotate were from the chloroplast. Genes predicted by MAKER-P that are not identified by YeATSAM are listed with their homology to corresponding genes in the TAIR database.

TRS	ORF	Len	TAIR	Description	E-value
C52274_G4_I1_B	189	231	ATCG00720.1	PETB photosynthetic electron transfer B	4.00-155
C52274_G4_I1_C	231	177	ATCG00730.1	PETD photosynthetic electron transfer D	1.00e-108
C53854_G1_I1_A	45	98	ATCG00070.1	PSBK photosystem II reaction center protein K precursor	1.00E-27
C53854_G1_I1_B	62	62	ATCG00080.1	PSBI photosystem II reaction center protein I	3.00E-20
C54343_G2_I1_A	8	91	ATCG00580.1	PSBE photosystem II reaction center protein E	4.00E-54
C59245_G1_I1_B	70	95	ATCG00710.1	PSBH photosystem II reaction center protein H	4.00E-43
WALNUT_00014004-RA	-	1117	AT5G16850.1	TERT Telomerase reverse transcriptase	0.0
WALNUT_00018632-RA	-	295	ATMG00560.1	RPL2 Nucleic acid-binding, OB-fold-like protein	9e-152
WALNUT_00019747-RA	-	326	AT1G24040.1	Acyl-CoA N-acyltransferases (NAT) superfamily protein	5e-121
WALNUT_00031866-RA	-	311	AT5G07810.1	SNF2 domain-containing protein/helicase domain- containing	9e-115
WALNUT_00020600-RA	-	155	ATCG01240.1	RPS7.2 ribosomal protein S7 chrC:140704-141171	1e-108
WALNUT_00016414-RA	-	231	AT5G41850.1	alpha/beta-Hydrolases superfamily protein | chr5:16756698-16757791	6e-96
WALNUT_00027509-RA	-	289	AT2G43190.3	ribonuclease P family protein | chr2:17956220-17957833	2e-94
WALNUT_00022174-RA	-	389	AT2G07707.1	Plant mitochondrial ATPase, F0 complex, subunit	5e-86
WALNUT_00018616-RA	-	124	ATCG00890.1	NDHB.1 NADH-Ubiquinone/plastoquinone (complex I)	1e-79
WALNUT_00007302-RA	-	924	AT5G14990.1	BEST Arabidopsis thaliana protein match is: myosin	2e-79

Furthermore, MAKER-P failed to annotate any FAD-binding berberine bridge enzymes (BBE) in the WGS ( Kutchan & Dittrich, 1995). These enigmatic enzymes are implicated in the transformation of (S)-reticuline to (S)-scoulerine during benzophenanthridine alkaloid biosynthesis in plants ( Winkler *et al.*, 2006). This pathway is over-expressed upon osmotic stress and pathogen attack ( Attila *et al.*, 2008; González-Candelas *et al.*, 2010), provides resistance in lettuce, sunflower and transgenic tobacco by generating anti-microbial compounds ( Custers *et al.*, 2004), and has unknown functions at specific developmental stages in *Arabidopsis* ( Irshad *et al.*, 2008; Pagnussat *et al.*, 2005). Moreover, it is expressed in floral nectar (Nectarin V, *Nt*BBE) ( Carter & Thornburg, 2004) and roots of tobacco ( Kajikawa *et al.*, 2011), and in xylem sap of cabbage ( Ligat *et al.*, 2011) and grapevine ( Chakraborty *et al.*, 2016b). *Nt*BBE was constitutively expressed in the *Phytophthora infestans*-resistant potato genotype SW93-1015 ( Ali *et al.*, 2012). Benzophenanthridines are antimicrobial; the California poppy ( *Eschscholzia californica*) is used as a traditional medicine ( Cheney, 1963; Oldham *et al.*, 2010). Oral administration of the alkaloid berberine isolated from a Chinese herb lowered cholesterol in 32 hypercholesterolemic patients over three months ( Kong *et al.*, 2004). Berberine has also been shown to possess antidiabetic properties ( Lee *et al.*, 2006).

**Figure 3.**
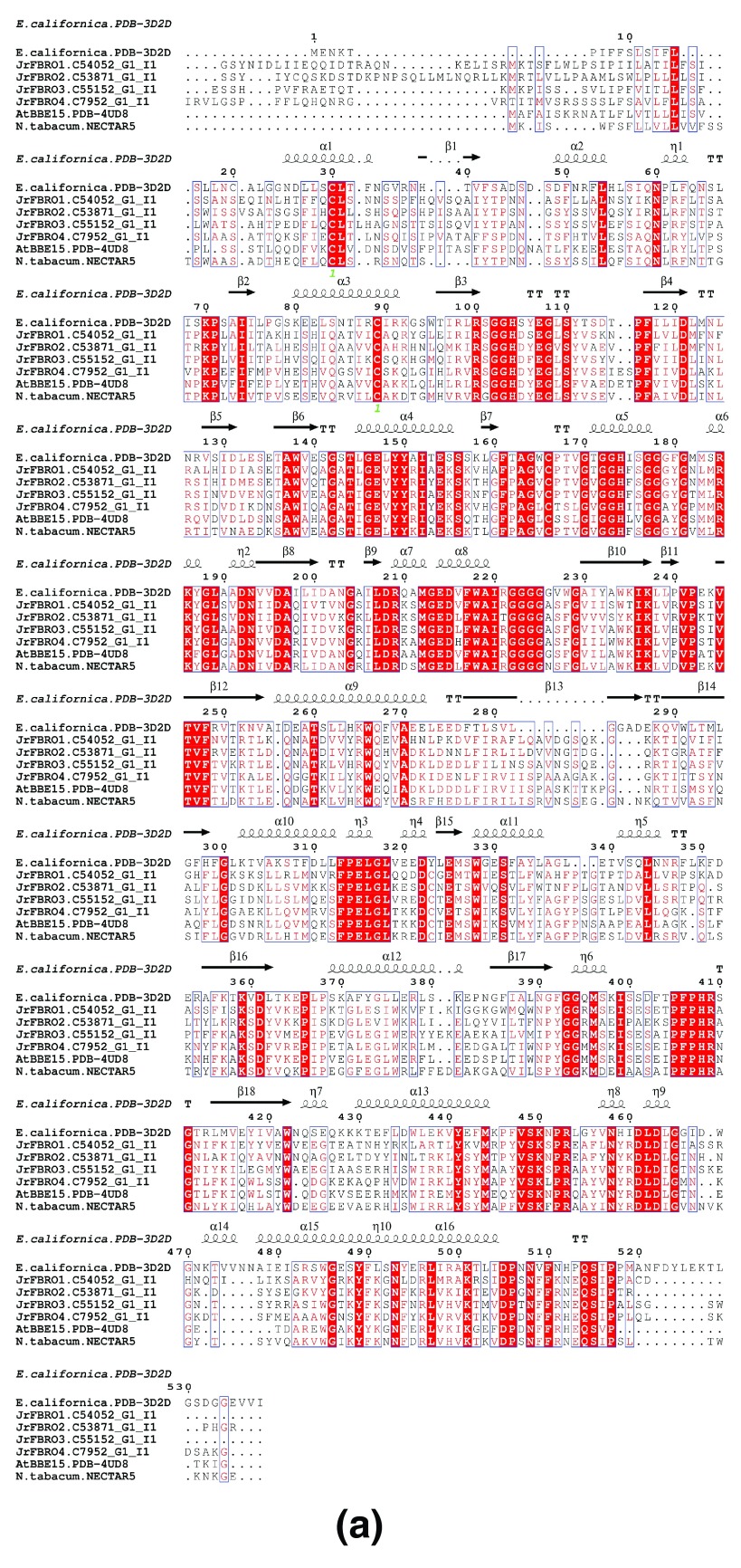

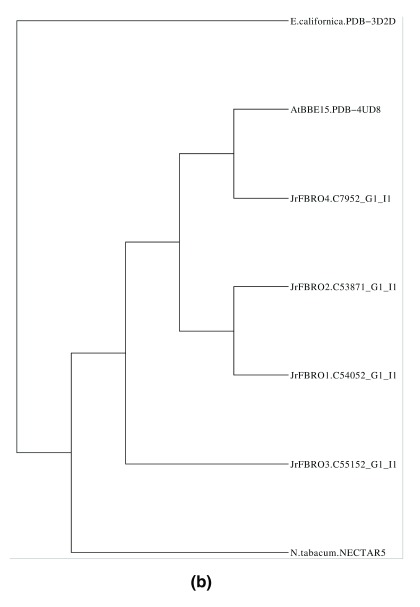
Multiple sequence alignment of BBE from walnut and other organisms. ( **a**) The JrBBE sequences were aligned to berberine bridge enzyme (BBE) genes from *Eschscholzia californica* (EcBBE; California poppy), *Arabidopsis thaliana* (AtBBE15) and *Nicotiana tabacum* (Nectarin V). Secondary structure information from the structure PDBid:3D2D ( *E. californica)* was used to annotate the sequences. The signal peptides are different in these proteins, suggesting different localization of these proteins in walnut. ( **b**) Phylogenetic tree generated from the multiple sequence alignment.

The number of BBE genes in different plant species varies significantly from one in moss ( *Physcomitrella patens*) to 64 in western poplar ( *Populus trichocarpa*) ( Daniel *et al.*, 2015). *A. thaliana* has 27 TAIR IDs assigned to BBE enzymes, with two splice variants ( Supplementary Table 1) ( Daniel *et al.*, 2015). Based on the current transcriptome, there are four full length BBE genes ( *Jr*BBE1 to 4) that map to different scaffolds in the WGS, in addition to other fragmented transcripts ( Table 2 and Table 3). *Jr*BBE1 (C54052_G1_I1) maps to the scaffold JCF7180001213852 and encodes a 564 aa long ORF, which has significant matches to Uniprot:P30986. The closest match of Uniprot:P30986 (with a low significance of 1E-07) to the MAKER-P annotation is ‘WALNUT 00019959-RA’, a 476 aa long cytokinin dehydrogenase. The sequence alignment of *Jr*BBE genes to Uniprot (P30986) is shown ( Figure 3a).

**Table 2.** FAD-binding berberine bridge enzymes (BBE) are undetected in MAKER-P. These oxidases are involved in the benzophenanthridine alkaloid biosynthesis in plants. *Arabidopsis* has 27 loci for this family (and a splice variant) ( Table 3). Here, there are four full length berberine bridge enzyme (BBE) genes (named *Jr*BBE1-4) identified using the transcriptome. Some of the proteins are truncated (like C54286_G1_I1), which might be an artifact of the Trinity assembler. Thus, this is not a complete enumeration of the *Jr*BBE genes.

Id	Transcript	Length	Scaffold	ORF	TAIR Id
*Jr*BBE1	C54052_G1_I1	564	JCF7180001213852	34	AT1G26420.1
*Jr*BBE2	C53871_G1_I1	564	JCF7180001217410	28	AT1G30700.1
*Jr*BBE3	C55152_G1_I1	552	JCF7180001222284:2429142-2890931	37	AT4G20820.1
*Jr*BBE4	C7952_G1_I1	559	JCF7180001218369	110	AT2G34790.1
	C54286_G2_I1	307	JCF7180001217076	35	AT1G11770.1
	C54286_G1_I1	128	JCF7180001217076	7	AT4G20830.1
	C12765_G1_I1	114	JCF7180001218369	8	AT4G20840.1
	C51815_G1_I4	168	JCF7180001218369	29	AT4G20860.1

**Table 3.** Expression counts (normalized) of transcripts from the FAD-binding berberine bridge enzyme (BBE) family. The genes have tissue-specific expression - *Jr*BBE3 is highly expressed in the roots and transition zone. The tissue abbreviations are from Chakraborty *et al.*, 2016a.

id	Transcript	CE	CI	CK	EM	FL	HC	HL	HP	HU	IF	LE	LM	LY	PK	PL	PT	RT	SE	TZ
*Jr*BBE1	C54052_G1_I1										44				4	136	197			
*Jr*BBE2	C53871_G1_I1	2		3		2			1	1			15					79	1	
*Jr*BBE3	C55152_G1_I1	43	34	25		62						1	2		35			1040		346
*Jr*BBE4	C7952_G1_I1	32	85	8		55	11	711	15	8	241	137	37	123	315	420	160	217	5	18
	C54286_G2_I1					33						20		30						
	C54286_G1_I1					19						7		24						
	C12765_G1_I1	26	77	2		39	4	42	8	23	23	19	5	22	9	2	8	6		
	C51815_G1_I4																			

As with the walnut transcriptome, the chickpea transcriptome (transHybrid.fasta: n=34760) ( Garg *et al.*, 2011) was split into three ORFs, each of which was BLAST’ed to the subset of plant proteins created from the Ensembl database. Subsequently, the ORFs with significant homology to this database (n=29263) were BLAST’ed to the set of annotated chickpea proteins in the NCBI database (n=34198). Most of these annotations were done using Gnomon ( Souvorov *et al.*, 2010) ( http://www.ncbi.nlm.nih.gov/bioproject/PRJNA190909), which analyzed ~35000 transcripts. There are ~1500 proteins identified by YeATSAM that are absent in the NCBI database (Evalue cutoff 1E-10). Some of these proteins and their corresponding genes in the TAIR database are shown ( Table 4). TC00902 is an interesting example with two merged genes: a hydrogen ion-transporting ATP synthase (TAIR ID: ATMG00640.1) and a cytochrome C biogenesis (TAIR ID: ATMG00900.1). While Gnomon identified the cytochrome C biogenesis protein (Genbank: XP_004500083.1), it failed to identify the ATP synthase. Unlike MAKER-P, Gnomon generates transcripts through predictive algorithms and does not take the transcriptome as an input. Notwithstanding, these chickpea genes remain unannotated despite the presence of a straightforward method to detect them from available transcripts.

**Table 4.** Selected genes in chickpea that are not annotated in the NCBI database. Most of the NCBI genes were predicted using Gnomon. YeATSAM used the publicly available transcriptome from chickpea to identify these genes. The corresponding genes from the TAIR database are shown. Several transcripts (like TC20962) encode multiple genes, while others (like TC01181) have only one significant ORF. TRid, transcript id; TAIRid: *Arabidopsis thaliana* id.

TRid	TAIRid	Description	Evalue
TC20962 A	ATMG00070.1	NAD9 NADH dehydrogenase subunit 9 chrM:23663-24235	3e-116
TC20962 B	AT2G07687.1	Cytochrome c oxidase, subunit III chr2:3311854-3312651	3e-107
TC20962 C	AT2G07674.1	Unknown conserved protein chr2:3269151-3269906	6e-41
TC01181	ATMG01360.1	COX1 cytochrome oxidase chrM:349830-351413	0.0
TC11063	AT3G30841.1	Cofactor-independent phosphoglycerate mutase chr3:12591595-12593401	0.0
TC06038	ATMG00090.1	Structural constituent of ribosome;protein binding chrM:25482-28733	3e-124
TC13206	AT3G13440.1	S-adenosyl-L-methionine-dependent methyltransferases superfamily	1e-118
TC07586	AT2G07725.1	Ribosomal L5P family protein chr2:3448402-3448959	2e-113
TC19047	ATMG00570.1	Sec-independent periplasmic protein translocase	8e-107
TC00902 B	ATMG00640.1	Hydrogen ion transporting ATP synthases, rotational	3e-104
TC15163	AT4G28360.1	Ribosomal protein L22p/L17e family protein chr4:14029294-14030926	1e-100
TC13677	AT5G05210.1	Surfeit locus protein 6 chr5:1548198-1549534	9e-91
TC13780 A	AT2G07707.1	Plant mitochondrial ATPase, F0 complex, subunit 8 protein	2e-90
TC18786	AT1G73440.1	Calmodulin-related chr1:27611418-27612182	5e-45

### Future work

Among the ~700 genes not detected by YeATSAM, there are ~500 genes with no matches in the complete ‘nr’ database. Of these, ~300 have no transcripts (SetA), while the remaining ~200 have matches among the transcripts (SetB). Considering the sensitivity of RNA-seq and the wide coverage of twenty tissues, it is a definite possibility that SetA are pseudogenes. Future work in YeATSAM will focus on methods to distinguish these two classes of genes.

### Conclusions

The availability of a RNA-seq-derived transcriptome from a newly sequenced organism like walnut, for which there are related annotated genomes ( *Arabidopsis*, *Vitis*, etc), immensely simplifies annotation of the genome and influences the choice of annotation software. Here, we introduce a new annotation method in the YeATS suite (YeATS Annotation Module - YeATSAM), which was used to annotate the newly-sequenced walnut genome using a simple workstation. The key differentiating factor in YeATSAM is the splitting of the assembled transcriptome into multiple ORFs ( Chakraborty *et al.*, 2015). Transcripts often have more than one significant ORF that must be handled differently depending on whether they map to the same or different proteins. We show that YeATSAM failed to annotate ~700 genes identified by MAKER-P, while identifying ~4000 genes missed by MAKER-P. While most of these genes have repetitive stretches, both methods missed vital genes identified by the other. Since many of the additional genes identified by MAKER-P have no known transcripts, we posit that these were identified using *ab initio* methods. In the absence of such an *ab initio* module in YeATSAM, we propose a combined method using both MAKER-P and YeATSAM to annotate the WGS. YeATSAM was also applied to the chickpea transcriptome and identified ~1000 proteins that are not annotated in the NCBI database. This transcriptome was assembled using Newbler v2.3 ( Garg *et al.*, 2011) and most of the 34198 chickpea proteins in the NCBI database were annotated using Gnomon, the standard annotation tool ( http://www.ncbi.nlm.nih.gov/genome/guide/gnomon.shtml).

## Software availability

Latest source code: https://github.com/sanchak/YeATSAM

Archived source code at time of publication: DOI: 10.5281/zenodo.165992 ( Sanchak, 2016)

License: GNU General Public License

## Supplementary material

**Supplementary Table 1. FAD-binding (Berberine family) reticuline oxidases in *Arabidopsis*.** These proteins are involved in the benzophenanthridine alkaloid biosynthesis in plants. Benzophenanthridine is an anti-microbial metabolite. There are 27 loci in chromosomes 1,2,4 and 5, with one splice variant (AT4G20830) marked with an asterisk.

## References

[ref-1] Ali A Moushib LI Lenman M: Paranoid potato: phytophthora-resistant genotype shows constitutively activated defense. *Plant Signal Behav.* 2012;7(3):400–408. 10.4161/psb.19149 22476463 PMC3443922

[ref-2] Attila C Ueda A Cirillo SL: *Pseudomonas aeruginosa* PAO1 virulence factors and poplar tree response in the rhizosphere. *Microb Biotechnol.* 2008;1(1):17–29. 10.1111/j.1751-7915.2007.00002.x 21261818 PMC3864428

[ref-3] Camacho C Madden T Ma N: BLAST Command Line Applications User Manual.2013 Reference Source

[ref-4] Campbell MS Law M Holt C: Maker-P: a tool kit for the rapid creation, management, and quality control of plant genome annotations. *Plant Physiol.* 2014;164(3):513–524. 10.1104/pp.113.230144 24306534 PMC3912085

[ref-5] Carter CJ Thornburg RW: Tobacco nectarin V is a flavin-containing berberine bridge enzyme-like protein with glucose oxidase activity. *Plant Physiol.* 2004;134(1):460–469. 10.1104/pp.103.027482 14730073 PMC316325

[ref-6] Chakraborty S Britton M Martínez-García PJ: Deep RNA-seq profile reveals biodiversity, plant-microbe interactions and a large family of NBS-LRR resistance genes in walnut ( *Juglans regia*) tissues. *AMB Express.* 2016a;6(1):12. 10.1186/s13568-016-0182-3 26883051 PMC4755957

[ref-7] Chakraborty S Britton MT Wegrzyn JL: YeATS - a tool suite for analyzing RNA-seq derived transcriptome identifies a highly transcribed putative extensin in heartwood/sapwood transition zone in black walnut [version 2; referees: 3 approved]. *F1000Res.* 2015;4:155. 10.12688/f1000research.6617.2 26870317 PMC4732554

[ref-8] Chakraborty S Nascimento R Zaini PA: Sequence/structural analysis of xylem proteome emphasizes pathogenesis-related proteins, chitinases and *β*-1, 3-glucanases as key players in grapevine defense against *Xylella fastidiosa*. *PeerJ.* 2016b;4:e2007. 10.7717/peerj.2007 27257535 PMC4888286

[ref-9] Chang Z Li G Liu J: Bridger: a new framework for *de novo* transcriptome assembly using RNA-seq data. *Genome Biol.* 2015;16:30. 10.1186/s13059-015-0596-2 25723335 PMC4342890

[ref-10] Cheney RH: Therapeutic potential of *Eschscholtziae californicae* herb. *Q J Crude Drug Res.* 1963;3(3):413–416. 10.3109/13880206309082400

[ref-11] Chu HT Hsiao WW Chen JC: EBARDenovo: highly accurate *de novo* assembly of RNA-seq with efficient chimera-detection. *Bioinformatics.* 2013;29(8):1004–1010. 10.1093/bioinformatics/btt092 23457040

[ref-12] Custers JH Harrison SJ Sela-Buurlage MB: Isolation and characterisation of a class of carbohydrate oxidases from higher plants, with a role in active defence. *Plant J.* 2004;39(2):147–160. 10.1111/j.1365-313X.2004.02117.x 15225281

[ref-13] Daniel B Pavkov-Keller T Steiner B: Oxidation of monolignols by members of the berberine bridge enzyme family suggests a role in plant cell wall metabolism. *J Biol Chem.* 2015;290(30):18770–18781. 10.1074/jbc.M115.659631 26037923 PMC4513132

[ref-14] Flintoft L: Transcriptomics: digging deep with RNA-seq. *Nat Rev Genet.* 2008;9:568 10.1038/nrg2423

[ref-15] Fu L Niu B Zhu Z: CD-HIT: accelerated for clustering the next-generation sequencing data. *Bioinformatics.* 2012;28(23):3150–3152. 10.1093/bioinformatics/bts565 23060610 PMC3516142

[ref-16] Garg R Patel RK Tyagi AK: *De novo* assembly of chickpea transcriptome using short reads for gene discovery and marker identification. *DNA Res.* 2011;18(1):53–63. 10.1093/dnares/dsq028 21217129 PMC3041503

[ref-17] González-Candelas L Alamar S Sánchez-Torres P: A transcriptomic approach highlights induction of secondary metabolism in citrus fruit in response to *Penicillium digitatum* infection. *BMC Plant Biol.* 2010;10:194. 10.1186/1471-2229-10-194 20807411 PMC2956543

[ref-18] Grabherr MG Haas BJ Yassour M: Full-length transcriptome assembly from RNA-seq data without a reference genome. *Nat Biotechnol.* 2011;29(7):644–652. 10.1038/nbt.1883 21572440 PMC3571712

[ref-19] Guindon S Lethiec F Duroux P: PHYML Online--a web server for fast maximum likelihood-based phylogenetic inference. *Nucleic Acids Res.* 2005;33(Web Server issue):W557–559. 10.1093/nar/gki352 15980534 PMC1160113

[ref-20] Holt C Yandell M: Maker2: an annotation pipeline and genome-database management tool for second-generation genome projects. *BMC Bioinformatics.* 2011;12:491. 10.1186/1471-2105-12-491 22192575 PMC3280279

[ref-21] Irshad M Canut H Borderies G: A new picture of cell wall protein dynamics in elongating cells of *Arabidopsis thaliana*: Confirmed actors and newcomers. *BMC Plant Biol.* 2008;8:94. 10.1186/1471-2229-8-94 18796151 PMC2551616

[ref-22] Joshi N Fass J: Sickle: A sliding-window, adaptive, quality-based trimming tool for fastq files. (version 1.33) [software];2011 Reference Source

[ref-23] Jukanti AK Gaur PM Gowda CL: Nutritional quality and health benefits of chickpea ( *Cicer arietinum* L.): a review. *Br J Nutr.* 2012;108(Suppl 1):S11–S26. 10.1017/S0007114512000797 22916806

[ref-24] Kajikawa M Shoji T Kato A: Vacuole-localized berberine bridge enzyme-like proteins are required for a late step of nicotine biosynthesis in tobacco. *Plant Physiol.* 2011;155(4):2010–2022. 10.1104/pp.110.170878 21343426 PMC3091092

[ref-25] Kersey PJ Allen JE Armean I: Ensembl genomes 2016: more genomes, more complexity. *Nucleic Acids Res.* 2016;44(D1):D574–D580. 10.1093/nar/gkv1209 26578574 PMC4702859

[ref-26] Kinoshita A Nakamura Y Sasaki E: Gain-of-function phenotypes of chemically synthetic CLAVATA3/ESR-related (CLE) peptides in *Arabidopsis thaliana* and *Oryza sativa.* *Plant Cell Physiol.* 2007;48(12):1821–1825. 10.1093/pcp/pcm154 17991631

[ref-27] Kong W Wei J Abidi P: Berberine is a novel cholesterol-lowering drug working through a unique mechanism distinct from statins. *Nat Med.* 2004;10(12):1344–1351. 10.1038/nm1135 15531889

[ref-28] Kutchan TM Dittrich H: Characterization and mechanism of the berberine bridge enzyme, a covalently flavinylated oxidase of benzophenanthridine alkaloid biosynthesis in plants. *J Biol Chem.* 1995;270(41):24475–24481. 10.1074/jbc.270.41.24475 7592663

[ref-29] Larkin MA Blackshields G Brown NP: Clustal W and Clustal X version 2.0. *Bioinformatics.* 2007;23(21):2947–2948. 10.1093/bioinformatics/btm404 17846036

[ref-30] Law M Childs KL Campbell MS: Automated update, revision, and quality control of the maize genome annotations using MAKER-P improves the B73 refgen_v3 gene models and identifies new genes. *Plant Physiol.* 2015;167(1):25–39. 10.1104/pp.114.245027 25384563 PMC4280997

[ref-31] Lee YS Kim WS Kim KH: Berberine, a natural plant product, activates AMP-activated protein kinase with beneficial metabolic effects in diabetic and insulin-resistant states. *Diabetes.* 2006;55(8):2256–2264. 10.2337/db06-0006 16873688

[ref-32] Ligat L Lauber E Albenne C: Analysis of the xylem sap proteome of *Brassica oleracea* reveals a high content in secreted proteins. *Proteomics.* 2011;11(9):1798–1813. 10.1002/pmic.201000781 21413152

[ref-33] Lohse M Bolger MA Nagel A: *RobiNA*: a user-friendly, integrated software solution for RNA-Seq-based transcriptomics. *Nucleic Acids Res.* 2012;40(Web Server issue):W622–W627. 10.1093/nar/gks540 22684630 PMC3394330

[ref-34] Martínez-García PJ Crepeau MW Puiu D: The walnut ( *Juglans regia*) genome sequence reveals diversity in genes coding for the biosynthesis of non-structural polyphenols. *Plant J.* 2016;87(5):507–32. 10.1111/tpj.13207 27145194

[ref-35] Mbandi SK Hesse U van Heusden P: Inferring *bona fide* transfrags in RNA-Seq derived-transcriptome assemblies of non-model organisms. *BMC Bioinformatics.* 2015;16(1):58. 10.1186/s12859-015-0492-5 25880035 PMC4344733

[ref-36] Neale DB Wegrzyn JL Stevens KA: Decoding the massive genome of loblolly pine using haploid DNA and novel assembly strategies. *Genome Biol.* 2014;15(3):R59. 10.1186/gb-2014-15-3-r59 24647006 PMC4053751

[ref-37] Nelson N Yocum CF: Structure and function of photosystems I and II. *Annu Rev Plant Biol.* 2006;57:521–565. 10.1146/annurev.arplant.57.032905.105350 16669773

[ref-38] Oldham JT Hincapie M Rejtar T: Shotgun proteomic analysis of yeast-elicited California poppy ( *Eschscholzia californica*) suspension cultures producing enhanced levels of benzophenanthridine alkaloids. *J Proteome Res.* 2010;9(9):4337–4345. 10.1021/pr1000412 20690678

[ref-39] Pagnussat GC Yu HJ Ngo QA: Genetic and molecular identification of genes required for female gametophyte development and function in *Arabidopsis*. *Development.* 2005;132(3):603–614. 10.1242/dev.01595 15634699

[ref-40] Rice P Longden I Bleasby A: EMBOSS: the European Molecular Biology Open Software Suite. *Trends Genet.* 2000;16(6):276–277. 10.1016/S0168-9525(00)02024-2 10827456

[ref-41] Robert X Gouet P: Deciphering key features in protein structures with the new endscript server. *Nucleic Acids Res.* 2014;42(Web Server issue):W320–W324. 10.1093/nar/gku316 24753421 PMC4086106

[ref-42] Sanchak: sanchak/YeATSAM 1 [Data set]. *Zenodo.* 2016 Data Source

[ref-43] Schulz MH Zerbino DR Vingron M: *Oases*: robust *de novo* RNA-seq assembly across the dynamic range of expression levels. *Bioinformatics.* 2012;28(8):1086–1092. 10.1093/bioinformatics/bts094 22368243 PMC3324515

[ref-44] Shin LJ Lo JC Yeh KC: Copper chaperone antioxidant protein1 is essential for copper homeostasis. *Plant Physiol.* 2012;159(3):1099–1110. 10.1104/pp.112.195974 22555879 PMC3387697

[ref-45] Simpson JT Wong K Jackman SD: Abyss: a parallel assembler for short read sequence data. *Genome Res.* 2009;19(6):1117–1123. 10.1101/gr.089532.108 19251739 PMC2694472

[ref-46] Souvorov A Kapustin Y Kiryutin B: Gnomon-NCBI eukaryotic gene prediction tool. *NCBI.* 2010;1–24. Reference Source

[ref-47] Sprunck S Rademacher S Vogler F: Egg cell-secreted ec1 triggers sperm cell activation during double fertilization. *Science.* 2012;338(6110):1093–1097. 10.1126/science.1223944 23180860

[ref-48] Trapnell C Pachter L Salzberg SL: TopHat: discovering splice junctions with RNA-seq. *Bioinformatics.* 2009;25(9):1105–1111. 10.1093/bioinformatics/btp120 19289445 PMC2672628

[ref-49] Trapnell C Roberts A Goff L: Differential gene and transcript expression analysis of RNA-seq experiments with TopHat and cufflinks. *Nat Protoc.* 2012;7(3):562–578. 10.1038/nprot.2012.016 22383036 PMC3334321

[ref-50] Wang L Feng Z Wang X: DEGseq: an R package for identifying differentially expressed genes from RNA-seq data. *Bioinformatics.* 2010;26(1):136–138. 10.1093/bioinformatics/btp612 19855105

[ref-51] Wang Z Gerstein M Snyder M: RNA-seq: a revolutionary tool for transcriptomics. *Nat Rev Genet.* 2009;10(1):57–63. 10.1038/nrg2484 19015660 PMC2949280

[ref-52] Wilkins TA Smart LB: Isolation of RNA from plant tissue. Ed. Paul A. Kriedg, *A laboratory guide to RNA: Isolation, Analysis, and Synthesis* 1996;21–42. Reference Source

[ref-53] Winkler A Hartner F Kutchan TM: Biochemical evidence that berberine bridge enzyme belongs to a novel family of flavoproteins containing a bi-covalently attached fad cofactor. *J Biol Chem.* 2006;281(30):21276–21285. 10.1074/jbc.M603267200 16728404

[ref-54] Winkler A Lyskowski A Riedl S: A concerted mechanism for berberine bridge enzyme. *Nat Chem Biol.* 2008;4(12):739–741. 10.1038/nchembio.123 18953357

[ref-55] Yandell M Ence D: A beginner’s guide to eukaryotic genome annotation. *Nat Rev Genet.* 2012;13(5):329–342. 10.1038/nrg3174 22510764

[ref-56] Yu J Xue JH Zhou SL: New universal *matK* primers for DNA barcoding angiosperms. *J Syst Evol.* 2011;49(3):176–181. 10.1111/j.1759-6831.2011.00134.x

[ref-57] Zerbino DR Birney E: Velvet: algorithms for de novo short read assembly using de Bruijn graphs. *Genome Res.* 2008;18(5):821–829. 10.1101/gr.074492.107 18349386 PMC2336801

