# Supplementary material for: YeATSAM analysis of the walnut and chickpea transcriptome reveals key genes undetected by current annotation tools
Source: F1000Res. 2016 Nov 17;5:2689. [Version 1] doi: 10.12688/f1000research.10040.1 (PMC5200947; doi:10.12688/f1000research.10040.1)
Supplement: Supplementary file 1 [file f1000research-5-10816-s0000.tgz › 46deaef3-e2ce-443b-9b4c-724e2fb27af2.docx]

Supplementary Table. 1: **FAD-binding (Berberine family) reticuline oxidases** **in** *Arabidopsis*: These proteins are involved in the benzophenanthridine alkaloid biosynthesis in plants. Benzophenanthridine is an anti-microbial metabolite. There are 27 loci in chromosomes 1,2,4 and 5, with one splice variant (AT4G20830) marked with an asterisk.

| Chromosome | TAIRid | loci | Direction/Length |
| --- | --- | --- | --- |
| chr1 | AT1G01980.1 | chr1:340374-341999 | reverse-length=541 |
| chr1 | AT1G11770.1 | chr1:3975679-3977289 | forward-length=536 |
| chr1 | AT1G26380.1 | chr1:9126901-9128508 | reverse-length=535 |
| chr1 | AT1G26390.1 | chr1:9130164-9131756 | reverse-length=530 |
| chr1 | AT1G26400.1 | chr1:9133291-9134874 | reverse-length=527 |
| chr1 | AT1G26410.1 | chr1:9138774-9140432 | reverse-length=552 |
| chr1 | AT1G26420.1 | chr1:9141715-9143304 | reverse-length=529 |
| chr1 | AT1G30700.1 | chr1:10892623-10894437 | forward-length=527 |
| chr1 | AT1G30710.1 | chr1:10895280-10896875 | forward-length=531 |
| chr1 | AT1G30720.1 | chr1:10898197-10899780 | forward-length=527 |
| chr1 | AT1G30730.1 | chr1:10900854-10902434 | forward-length=526 |
| chr1 | AT1G30740.1 | chr1:10903029-10904630 | forward-length=533 |
| chr1 | AT1G30760.1 | chr1:10918321-10920441 | forward-length=534 |
| chr1 | AT1G34575.1 | chr1:12657149-12658732 | reverse-length=527 |
| chr2 | AT2G34790.1 | chr2:14673998-14677237 | reverse-length=532 |
| chr2 | AT2G34810.1 | chr2:14685292-14686914 | forward-length=540 |
| chr4 | AT4G20800.1 | chr4:11139656-11141242 | forward-length=528 |
| chr4 | AT4G20820.1 | chr4:11150160-11151758 | forward-length=532 |
| chr4 | *AT4G20830.1 | chr4:11155486-11157577 | forward-length=570 |
| chr4 | *AT4G20830.2 | chr4:11155486-11157108 | forward-length=540 |
| chr4 | AT4G20840.1 | chr4:11157916-11159535 | forward-length=539 |
| chr4 | AT4G20860.1 | chr4:11172726-11174318 | forward-length=530 |
| chr5 | AT5G44360.1 | chr5:17872100-17873698 | reverse-length=532 |
| chr5 | AT5G44380.1 | chr5:17878873-17881369 | reverse-length=541 |
| chr5 | AT5G44390.1 | chr5:17882329-17884906 | reverse-length=542 |
| chr5 | AT5G44400.1 | chr5:17886365-17888071 | reverse-length=537 |
| chr5 | AT5G44410.1 | chr5:17891246-17892853 | reverse-length=535 |
| chr5 | AT5G44440.1 | chr5:17910694-17912295 | reverse-length=533 |
