# Supplementary material for: YeATSAM analysis of the walnut and chickpea transcriptome reveals key genes undetected by current annotation tools
Source: F1000Res. 2016 Nov 17;5:2689. [Version 1] doi: 10.12688/f1000research.10040.1 (PMC5200947; doi:10.12688/f1000research.10040.1)
Supplement: Peer review history

# Peer review: Referee response for version 1

**DOI:** 10.5256/f1000research.10816.r18792

**Published:** 2016-12-28

**Authors:** Nic Herndon

**Affiliations:** 1 Department of Ecology and Evolutionary Biology, Plant Computational Genomics Lab, University of Connecticut, Storrs, CT, USA

The article presents an annotation method, YeATSAM, that leverages the information contained in RNA-Seq derived transcriptomes. The method was compared with two other annotation methods using two organisms: MAKER-P (a RNA evidence based and *ab initio* hybrid method) with walnut, and NCBI Gnomon (a homology based and *ab initio* hybrid method) with chickpea. Although YeATSAM and MAKER-P identified same genes, there were also genes that were identified by only one of them (about 4,000 by YeATSAM and about 700 by MAKER-P), as well as genes that both methods failed to identify. Similarly, YeATSAM identified about 1,000 genes that Gnomon failed to identify.

 The article is well written, the analysis is technically sound, the tables and figures present the results well, and the conclusions are supported by the data. Nonetheless, I would suggest the following changes:

- Address discrepancies in the numbers reported, e.g., 20 tissues (in abstract, introduction, results, and future work) v. 19 tissues (in methods: 15 samples + four additional samples); 700 (in results) v. ~700 (in future work, and coclusions); ~1,500 chickpea proteins (in results) v. ~1,000 (in conclusions). Instead of approximate values report actual values.
- As the tool is designed to be used with other organisms (besides walnut and chickpea), make the method and workflow (Figure 1) independent of any organism (e.g., the input to YeATSAM is the genome sequence rather than the walnut genome).
- Figure 3 (b) can be resized without losing its readability.

I have read this submission. I believe that I have an appropriate level of expertise to confirm that it is of an acceptable scientific standard.

# Peer review: Referee response for version 1

**DOI:** 10.5256/f1000research.10816.r17771

**Published:** 2016-12-06

**Authors:** Margaret E. Staton, Miriam Payá Milans

**Affiliations:** 1 Department of Entomology and Plant Pathology, University of Tennessee, Knoxville, TN, USA

This work focuses on a current major challenge in improving genome and transcriptome automated annotation. It also deals with difficulties derived from imperfect de novo assemblies, such as transcripts representing fused and split genes. The increasing affordability to generate sequencing data enhances the demand for more powerful annotation predicting tools and pipelines, although exact annotations will still remain for wet-lab experimentation. This paper compares the YeATSAM tool to previously annotated genomes, in which existing de novo assemblies are used as generated and analyzed with blast, interproscan or similar tools for homology-based annotation. Even though the paper indicates novelty of the method, there are critical points that need modification. 

 The method reported here - YeATSAM - is not clearly different from the work already reported in a previous paper ^1^. This method reported here (identify 3 longest ORFs, then blast to known proteins, then merge or split if needed) looks identical to previously published in F1000 Research ^1^ - for instance, Figure 1 in the previously published paper is an almost identical replica of the Figure 1 in this paper. The current work does appropriately cite this previous paper. However, if there is a novel algorithm to describe here, it needs to be clearly delineated from this previous work. Otherwise, it should just be cited.

 This previous publication also compares the annotation of the walnut genome by YeATS and Maker-P. The previous paper and this paper both profile walnut transcripts where ORFs were merged and transcripts that match multiple proteins; this paper does use different transcripts to demonstrate the methodology and results. To emphasize the novelty of the present paper, the authors should clarify exactly what this paper offers in addition to the previous paper. In this regard, the paper does go a bit further than the previous one by detailing genes that were unannotated by MAKER-P but found via this method; those genes were not reported previously. If the algorithm has not changed from the previous work, a new focus for this paper is needed, possibly reporting these novel genes such as the BBEs.

 The addition of the chickpea genome annotation is barely described - a single short results paragraph. The author also has an existing F1000 research article describing the use of YeATS on chickpea transcripts and describing the detection of missed genes and describing multiple ORFs mapping to different proteins and fragmented ORFs of the same protein ^2^. How does this report differ from that one? That one is not cited in this report

 Data reproducibility and accessibility - the new annotations are not made available for either walnut or chickpea (unless they are the same as the ones provided already in Chakraborty et al. 2015 ^1^). It would be very difficult to replicate this experiment. No parameters or commands are provided to determine how PHYML, ClustalW or ENDscript server were utilized. I confirmed that YeATSAM.zip (listed as YeATSAM.tgz in manuscript) with README is available for download and the links to data are functional. I was unable to install YeATSAM; the installation and usage instructions are very vague. 

 Specifics:

 The joined results of MAKER-P and YeATSAM look promising for improving genome annotations. However, a figure or table describing the total number of genes predicted by each software and the overlap would be very helpful to visualize the results. The report commonly has words like “several” or “many”  and the usage of “~” in front of numbers. Numbers should be reported exactly where they are important to the method and results. Examples:

- “A BLAST database of protein peptides (plantpep.fasta: 1M seqeunces) using ~30 organisms (list.plants)” - also list.plants does not link to anything.
- “About 3% of transcripts have ORFs that map to different proteins”
- “MAKER-P and YeATSAM each failed to annotate several proteins identified by the other (MAKER-P=~4000; YeATSAM=700)”
- “Among the ~700 genes not detected by YeATSAM, there are ~500 genes with no matches in the complete ‘nr’ database. Of these, ~300 have no transcripts (SetA), while the remaining ~200 have matches among the transcripts (SetB).”

Based on the content of the manuscript, the introduction focuses adequately on the explanation of the problematic of annotating newly assembled genomes and transcriptomes. However, a deeper introduction to the software utilized may be relevant for a better understanding of their choice and also of their basic mechanics. In relation to the results commented on the introduction, the relevance of some of the selected genes is not clear. Specifically, the relevance of the three “critical” proteins not detected by YeATSAM, which are not transcribed and are thus considered pseudogenes, is confusing.

 In relation to the generation of de novo assemblies, the authors are suggested to provide detail on how the assemblies were combined, considering that the simple addition of libraries would lead to high redundancy. For the walnut genome, were the MAKER-P and YeATSAM packages using the same set of RNASeq reads? This would be an important point to emphasize - a true comparison of the two methods would preferably use the same starting point. The original walnut paper reports using 19 libraries (Martinez-Garcia et al. 2016); this paper reports 20 libraries. 

 In the results and discussion section, the manipulation of ORFs is an interesting concept, although the difference to the described methodology in Chakraborty et al. 2015 ^1^ is not clear. The use of the term ORF is confusing here since it appears that the merged sequences are the encoded peptides, while ORFs are nucleotide sequences. Moreover, it seems likely the ORFs from the same gene might match different proteins because they are being compared to 30 different organisms. The ORFs could match to the orthologs of the gene in question from different organisms. (i.e. they have different matches to database entries, both orthologs, but they are legitimately from the same gene). In this case, merging is the best avenue, but the software would actually split the transcript apart. Was this seen in some transcripts? Also, when referring to significance with similar proteins, values should be provided.

 The authors mention that many genes unannotated by MAKER-P have repetitive stretches. What types of repetitive stretches? There is no methodology given for this analysis? This needs to be described/explained. In regards to the sentence “Although most of these unannotated proteins have repetitive sequences (transposable elements)” - does that mean the unannotated proteins originate from within transposable elements, or transposable elements have inserted into the gene itself? 

 The authors do not address the overall differences in proteins detected by each annotation program - is a pattern that may explain these? Pseudogenes are mentioned twice, but this idea is not fully explained. While 20 tissues will capture many genes, it is probably not exhaustive – is there any additional evidence these “genes” are actually pseudogenes such as premature stop codons or frameshift mutations?

 Minor:

 This sentence needs improved clarity:

 “The BLAST results of list.transcriptome.clean.ORFS: 320K on ‘plantpep.fasta’ was processed using a cutoff: bitscore=60, Evalue~=1E-10"

 Heading “Transcripts with multiple ORFs” - the section above also deals with transcripts with multiple ORFs. This heading could be clarified.

 Some revision on the writing would improve readability. Abbreviations are recommended to be properly specified at first use in the manuscript and always in figures. Also, numbers and units should be spaced. In relation to the language, the authors are advised to review the use of scientific English, as well as verb tense consistency. 

 In Table 1, the main line indicates proteins not annotated by either program while the last line indicates listing of genes predicted by MAKER-P. These two sentences in the same caption lead to confusion. In addition, sizing and description of other figures might be improved.

We have read this submission. We believe that we have an appropriate level of expertise to state that we do not consider it to be of an acceptable scientific standard, for reasons outlined above.

## References

[1] ChakrabortySBrittonMWegrzynJButterfieldTMartínez-GarcíaPJReaganRLRaoBJLeslieCAAradhayaMNealeDWoesteKDandekarAM: YeATS - a tool suite for analyzing RNA-seq derived transcriptome identifies a highly transcribed putative extensin in heartwood/sapwood transition zone in black walnut. *F1000Res*.2015;4: 10.12688/f1000research.6617.2 155 10.12688/f1000research.6617.2 26870317PMC4732554

[2] ChakrabortyS: RNA-seq assembler artifacts can bias expression counts and differential expression analysis - case study on the chickpea transcriptome emphasizes importance of freely accessible data for reproducibility. *F1000Research*.2016;5: 10.12688/f1000research.9667.2 10.12688/f1000research.9667.2

# Peer review: Referee response for version 1

**DOI:** 10.5256/f1000research.10816.r17772

**Published:** 2016-11-30

**Authors:** Carmen Cuadrado, Africa Sanchiz

**Affiliations:** 1 Department of Food Technology, SGIT - INIA (Deputy Directorate General of Research and Technology-National Institute for Agricultural and Food Research and Technology), Madrid, Spain; 2 Departamento de Tecnología de Alimentos, SGIT-INIA, Madrid, Spain

In this paper the authors investigate a new annotation method in the YeATS suite (YeATS Annotation Module - YeATSAM), which was used to annotate the newly-sequenced walnut genome using a simple workstation. In YeATSAM the assembled transcriptome is splitting into multiple ORFs. They show that YeATSAM failed to annotate ~700 genes identified by MAKER-P, while identifying ~4000 genes missed by MAKER-P. While most of these genes have repetitive stretches, both methods missed important genes identified by the other. Since many of the additional genes identified by MAKER-P have no known transcripts, the authors suggest that these were identified using ab initio methods. In the absence of such an ab initio module in YeATSAM, they propose a combined method using both MAKER-P and YeATSAM to annotate the WGS. This work is very interesting because the results probe the adequacy of this new annotation method. In general, the presentation is clear and the conclusions are adjusted to the results obtained. The figures and tables are also clear.  Some comments are listed below:

  

- In the abstract, please change the order in “Results and Conclusions” part, from lines 17 to 21. Consider to mention first “YeATSAM used a […] chickpea transcriptome assembled using Newbler v2.3” and then that “1000 genes were identified, which were not previously annotated by Gnomon annotation tool”.
- Fourth and fifth paragraphs of Introduction could be changed to the discussion and in the introduction leave some short sentences about this.
- En fifth line of Methods section correct “seqeunces”.
- Please consider to explain further section “future work”.

We have read this submission. We believe that we have an appropriate level of expertise to confirm that it is of an acceptable scientific standard.
